# Rare disease knowledge enrichment through a data-driven approach

**DOI:** 10.1186/s12911-019-0752-9

**Published:** 2019-02-14

**Authors:** Feichen Shen, Yiqing Zhao, Liwei Wang, Majid Rastegar Mojarad, Yanshan Wang, Sijia Liu, Hongfang Liu

**Affiliations:** Department of Health Sciences Research, Mayo Clinic, 205 3rd Ave SW, Rochester, MN 55905 USA

**Keywords:** Data-driven approach, Rare disease, Knowledge enrichment, Differential diagnosis

## Abstract

**Background:**

Existing resources to assist the diagnosis of rare diseases are usually curated from the literature that can be limited for clinical use. It often takes substantial effort before the suspicion of a rare disease is even raised to utilize those resources. The primary goal of this study was to apply a data-driven approach to enrich existing rare disease resources by mining phenotype-disease associations from electronic medical record (EMR).

**Methods:**

We first applied association rule mining algorithms on EMR to extract significant phenotype-disease associations and enriched existing rare disease resources (Human Phenotype Ontology and Orphanet (HPO-Orphanet)). We generated phenotype-disease bipartite graphs for HPO-Orphanet, EMR, and enriched knowledge base HPO-Orphanet + and conducted a case study on Hodgkin lymphoma to compare performance on differential diagnosis among these three graphs.

**Results:**

We used disease-disease similarity generated by the eRAM, an existing rare disease encyclopedia, as a gold standard to compare the three graphs with sensitivity and specificity as (0.17, 0.36, 0.46) and (0.52, 0.47, 0.51) for three graphs respectively. We also compared the top 15 diseases generated by the HPO-Orphanet + graph with eRAM and another clinical diagnostic tool, the Phenomizer.

**Conclusions:**

Per our evaluation results, our approach was able to enrich existing rare disease knowledge resources with phenotype-disease associations from EMR and thus support rare disease differential diagnosis.

**Electronic supplementary material:**

The online version of this article (10.1186/s12911-019-0752-9) contains supplementary material, which is available to authorized users.

## Background

Rare diseases, although individually rare, collectively affect one in ten Americans. Approximately 7000 rare diseases exist, with more being discovered each year [1]. Patients with rare diseases face diagnostic delay: 40% of rare disease patients are diagnosed incorrectly before reaching a final diagnosis, of which 25% spend between 5 to 30 years on a chaotic journey through numerous referrals, investigations, and disease evolutions from early symptoms to a confirmatory diagnosis of their disease [2]. Although there are many genetic tests available for delivering precision medicine, how to identify patients who may benefit from those genetic tests is not obvious. Many rare diseases can be misdiagnosed as common diseases due to their rarity. It often takes substantial clinical time and effort before a rare disease is even a suspected diagnosis [3].

The diagnosis pathway of rare diseases is highly dependent on the associated clinical phenotypes, i.e., the observable characteristics, at the physical, morphologic, or biochemical level, of an individual [4]. Symptoms could be treated as phenotypes in symptomatic diagnosis. Taking *Hodgkin lymphoma* as an example, since symptoms of *Hodgkin lymphoma* are very similar to other diseases or conditions, such as *Cytomegalovirus*, *Sarcoidosis*, and *Toxoplasmosis* [5], it is meaningful to use underlying disease-phenotype associations to accelerate early differential diagnosis and largely shorten the diagnostic odyssey for patients.

Rare disease knowledge resources exist to assist the diagnosis of rare diseases. For example, the Genetic and Rare Diseases (GARD) resource provides curated information for more than 4700 rare diseases, including their symptoms, causes, inheritance, treatments, and prognoses as well as the latest research [6]. Orphanet [7] provides an expert-vetted and up-to-date encyclopedia of rare diseases along with their associated genes. The Human Phenotype Ontology (HPO) [8] provides a controlled vocabulary for clinical phenotypes by mining and integrating clinical phenotype knowledge from literature and a variety of rare disease resources.

Some other existing studies investigated the mining of associations between diseases and genes. For example, Zhang et al. combined the Latent Dirichlet Allocation (LDA) [9] with network-based computational approach [10] to discover disease-gene associations from large amount of PubMed literature [11]. Piro et al. developed a classification approach to predict disease-gene associations [12]. By leveraging a network distance measure and a random walk algorithm, Kohler et al. presented a method to prioritize candidate genes for hereditary disorders [13]. However, all of these studies focused solely on extracting information from literature or knowledge bases. It often takes substantial time and effort before the suspicion of a rare disease is even raised to utilize those resources due to its rarity.

There are some related studies utilizing either electronic medical record (EMR) or literature or both to investigate diseases, phenotypes and their associations. For example, Xu et al. introduced text mining result of disease-phenotype associations by analyzing sentences from MEDLINE [14]. In another study, Garcelon et al. described a text mining based analysis leveraging TF-IDF to discover associations between clinical phenotypes and rare diseases [15]. Their results showed that phenotypes identified in EMR can be a useful source of evidence to provide rare disease specialists with candidate phenotypes. The eRAM is an encyclopedia of rare disease annotations mined from 10 million scientific publications and EMR [16]. Authors of the eRAM implemented a web-based tool to provide clinicians with next-step information of disease-disease associations in addition to disease-phenotype associations. The tool systematically incorporates disease-phenotype associations of rare diseases from both published medical literatures and clinical data. Hassan et al. investigated on extracting associations between rare diseases and phenotypes to enrich existing ontology [17]. The Phenomizer [18] is a clinical diagnostic tool that aims to help clinicians to identify the potential diagnostic candidates. It is built based on the HPO, Orphanet and Online Mendelian Inheritance in Man (OMIM) [19]. Unfortunately, EMR was not incorporated in [17, 18].

Here, we used the HPO annotation file named “phenotype_annotation.tab” accessed in July 2017 for association information between HPO terms and rare diseases in Orphanet [20]. These associations, which we referred to as HPO-Orphanet associations, were treated as rare disease knowledge resource in this study. We propose to enrich the HPO-Orphanet through mining association information between clinical phenotypes and diseases using EMR. Such enriched information, named as HPO-Orphanet+, can be used to link similar rare/common diseases and provide differential diagnostic decision aid at the point of care for rare disease diagnosis.

In the following, we first introduce the methods used in our study. We then describe our experimental evaluations. Results are presented next followed by discussion. We conclude our study with potential future work.

## Methods

### Materials

All clinical notes during the years of 2010 to 2015 from Mayo Clinic EMR were used for the study, including Consultant Notes (CON), Subsequent Visit Notes (SV), Emergency Medicine Notes (EMV), Hospital Admission Notes (ADM), and so on. For each note type, we focused on the diagnosis section of the notes which summarizes problems for each patient. The resulting corpus contains 12.8 million clinical notes corresponding to 729,000 patients. In our previous work, we have developed a phenotype-disease annotation pipeline that utilized the HPO and the Unified Medical Language System (UMLS) [21] to extract phenotypic and disease terms from clinical narratives [22, 23], where disease and phenotype appeared in the same clinical note was considered to be a phenotype-disease association. Specifically, the HPO was used to identify rare diseases and their phenotypic characterization mentioned in clinical narratives, and the UMLS was utilized to detect synonyms for any phenotypic terms. We limited our annotation to sections containing problems and diagnoses where 38,097 patients were found to have at least one diagnosis of a rare disease. Leveraging this pipeline, we extracted 2808 unique phenotypes from notes and 9,292,969 phenotype-disease associations in total, from which 164,792 associations were related to 1449 rare diseases and the rest were generated from 13,821 common diseases.

Semantic MEDLINE Database (SemMedDB), a repository of semantic predications extracted from the titles and abstracts of all PubMed citations [24–27], was used in this study to quantify the explanatory power of enriched associations. We used SemMedDB Version 25 in this study.

We used the eRAM to build a gold standard on disease differential diagnosis and used the gold standard to evaluate performance among three bipartite graphs. In addition, we compared the top 15 differential diagnostic candidates generated by the HPO-Orphanet+, Phenomizer, and eRAM. Specifically, the HPO-Orphanet+ and eRAM ranked diagnostic candidates by the descending order of Jaccard similarity score [28], and the Phenomizer ranked diagnostic candidates by the descending order of Information Content (IC)-based similarity score proposed in [18].

### System design

**Fig. 1:**
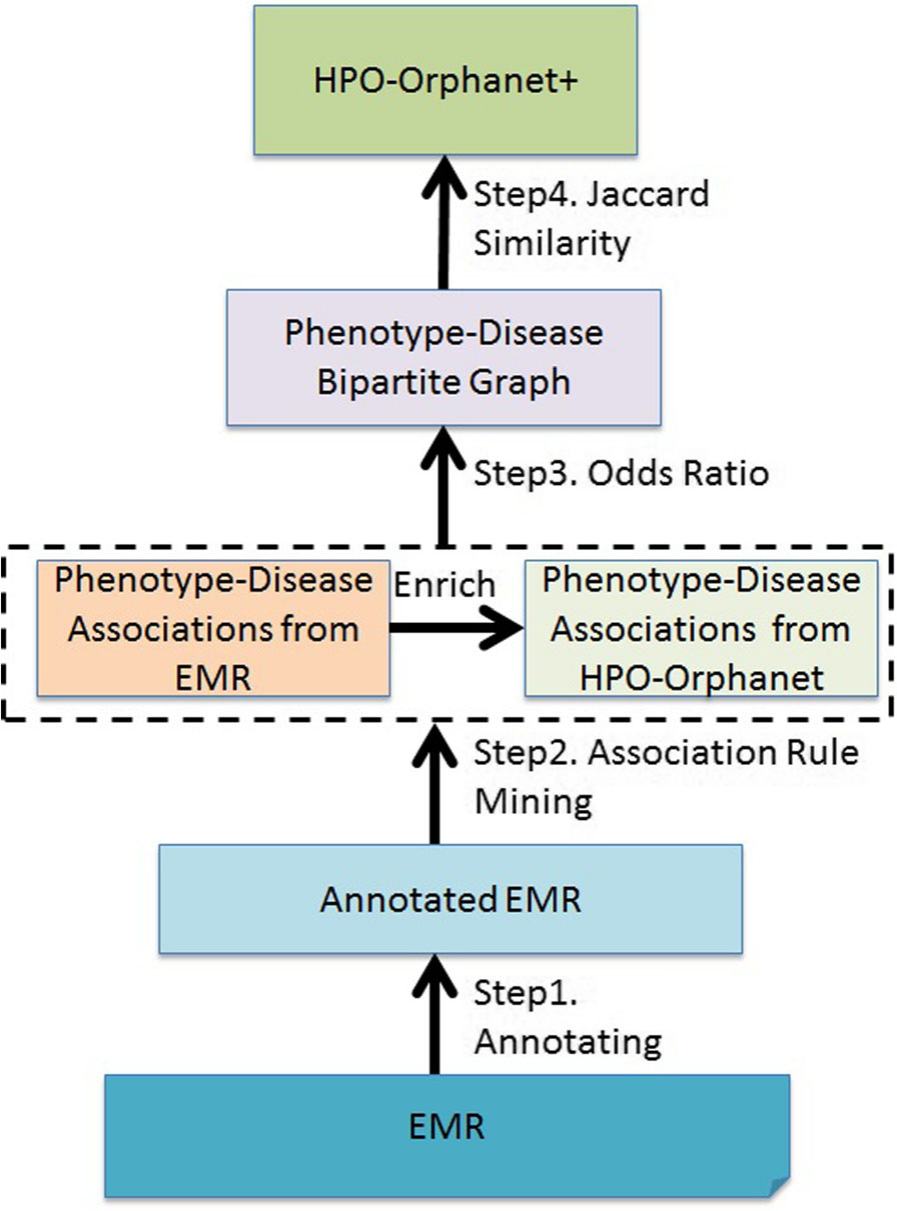
System workflow

The overall workflow of our study is shown in Fig. 1. After obtaining raw phenotype-disease pairs from EMR using the previously developed annotation pipeline, we formulated the task of mining association information between clinical phenotypes and diseases as an association rule mining task [29]. Specifically, patients are considered as transactions and their phenotypes and diseases are considered as items. The phenotype-disease association discovery can be defined as one item rule {Phenotype} ➔ {Disease}. We leveraged the support and confidence metrics (Eqs. 1 and 2) to measure the importance of discovered phenotype-disease relationships [30].

Given phenotype P and disease D, support is calculated as:

1 $$ {\displaystyle \begin{array}{c} support(P)=\frac{\#\mathrm{of}\ \mathrm{unique}\ \mathrm{patients}\ \mathrm{having}\ \mathrm{Phenotype}\ P\ }{\#\mathrm{of}\ \mathrm{unique}\ \mathrm{patients}\ }\\ {} support(D)=\frac{\#\mathrm{of}\ \mathrm{unique}\ \mathrm{patients}\ \mathrm{having}\ \mathrm{Disease}\ D}{\#\mathrm{of}\ \mathrm{unique}\ \mathrm{patients}\ }\\ {} support(PD)=\frac{\#\mathrm{of}\ \mathrm{unique}\ \mathrm{patients}\ \mathrm{having}\ \mathrm{Phenotype}\ P\&\mathrm{Disease}\ \mathrm{D}\ }{\#\mathrm{of}\ \mathrm{unique}\ \mathrm{patients}\ }\end{array}} $$

confidence is calculated as:

2 $$ confidence\left(P\Rightarrow D\right)=\frac{\mathrm{support}\ \left(\mathrm{P}\mathrm{D}\right)\ }{\mathrm{support}\ \left(\mathrm{P}\right)\ } $$

In addition, we filtered out less important phenotype-disease associations. In our previous study [31], we have demonstrated the use of odds ratio [32, 33] to detect significant phenotype-disease associations from a huge number of patient data, therefore, in this study, the odds ratio was applied on any disease-phenotype associations to find significant phenotypes for diseases. For any pair of disease D and phenotype P, the odds ratio OR(D, P) is defined as:

3 $$ OR\ \left(D,P\right)=\frac{\left(\# associations\ contain\ both\ D\  and\ P\right)\ast \left(\# associations\ contain\ neither\ D\  nor\ P\right)}{\left(\# associations\ contain\ D\  and\ phenotypes\ except\ P\right)\ast \left(\# associations\ contain\ P\  and\ diseases\ except\ D\right)} $$

In this study, we used a bipartite graph to represent associations between diseases and phenotypes. Given two disjoint and independent sets U and V, let U denote disease sets and V denote phenotype sets, the bipartite graph G is defined as a graph such that each edge connects a vertex in U to one in V [34].

Given a collection of phenotype-disease associations C, we implemented a heuristics for generating differential diagnostic candidates. For a disease D, we first selected those phenotypes with the corresponding lower bound of odds ratio values larger than one [35]. A phenotype-disease bipartite graph was then generated. Jaccard similarity was commonly adopted on detecting disease similarity [28], hence in this study, we applied Jaccard similarity on disease level, aiming to measure the similarity among diseases based on significant phenotypic features selected by odds ratio. Given two diseases D_i_ and D_j_, denoting phenotype sets for D_i_ and D_j_ as {P_i_} and {P_j_} respectively, the Jaccard similarity J (D_i_, D_j_) is defined as:

4 $$ J\left({D}_i,{D}_j\right)=\frac{\mid \left\{{P}_i\right\}\cap \left\{{P}_j\right\}\mid }{\left|\Big\{{P}_i\right\}\cup \left\{{P}_j\right\}\mid } $$

The derived HPO-Orphanet+ graph stored diseases as nodes and weighted edges as Jaccard similarity scores between diseases.

## Results

### Evaluation approach

Evaluation of our system comprised of three major components: 1) Enrichment of phenotype-disease associations, 2) Bipartite graph comparison, and 3) Performance on rare disease differential diagnosis.

#### Enrichment of phenotype-disease associations

We used phenotype-disease associations reported in the SemMedDB to quantify the explanatory power of enriched associations mined from EMR. We set average support and average confidence as minimum thresholds to pick top associations. We leveraged the increment of explanatory power (IEP) [36] to quantify the enrichment on HPO-Orphanet.

#### Increment of explanatory power (IEP)

We used explanatory power (EP) as defined in the study [36] to represent the associations explained by HPO-Orphanet:

5 $$ EP=\# of\ associations\ explained\  by\  knowledge\ base $$

We then quantified the knowledge increment by finding the increment of explanatory power (IEP) [36] for the enriched knowledge base generated by our approach compared to the initial HPO-Orphanet annotations:

6 $$ IEP=\frac{UK_i-{UK}_n}{UK_i} $$

where UK_i_ is the number of unexplained associations from the initial HPO-Orphanet knowledge and UK_n_ is the number of unexplained associations in the enriched knowledge resource HPO-Orphanet+.

#### Bipartite graph comparison

We limited our associations to diseases appearing in both EMR and HPO-Orphanet. We compared graph features for three bipartite graphs based on associations mined from EMR alone, HPO-Orphanet alone, and the combination of the prior two, HPO-Orphanet+. Graph characterization measurements we used were defined as follows:

#### Density

For undirected simple graphs, the graph density was defined as [37].

7 $$ Density(G)=\frac{2\left|E\right|}{\left|V\right|\left(\left|V\right|-1\right)} $$

where E is the number of edges in the graph and V is the number of vertices in the graph.

#### Average degree

The average degree of a vertex of a graph is the average number of edges connected to the vertex [38], and is defined as follows:

8 $$ \overline{\Delta }(G)=\frac{\sum \deg (v)}{\left|V\right|}=\frac{2\mid E\mid }{\left|V\right|} $$

where E is the number of edges in the graph and V is the number of vertices in the graph.

#### Performance on rare disease differential diagnosis

We compared performance for generating differential diagnostic candidates among HPO-Orphanet graph, EMR graph, and HPO-Orphanet+ graph.

To prepare the experiment, for any disease to be tested, we used the three aforementioned graphs to rank suggested diseases with descending order of Jaccard similarity score. We combined two disease-phenotype association files namely “eRAM Integrated Phenotype.txt” and “eRAM Integrated Symptom.txt” provided by the eRAM. Since those two files were manually annotated by domain experts, in this study, we considered the associations provided by the merged files as correct ones to prepare the gold standard. Based on 5356 curated diseases and their associated phenotypes/symptoms obtained from the files, we calculated Jaccard similarity score between each pair of diseases using Eq. 4. Such disease-disease similarity was used as a gold standard on differential diagnosis. In this evaluation, we first validated diagnostic candidates generated by the three bipartite graphs using the gold standard. We then compared the top 15 differential diagnostic candidates generated by the HPO-Orphanet+ graph, Phenomizer, and eRAM.

Table 1 shows the confusion matrix for performance evaluation. For any given tested disease, sensitivity and specificity were defined as shown in Eqs. 9 and 10.

9 $$ Sensitivity=\frac{TP}{TP+ FN} $$

10 $$ Specificity=\frac{TN}{TN+ FP} $$

**Table 1:** Confusion matrix for performance evaluation

	Differential diagnosis candidates in the eRAM gold standard	Differential diagnosis candidates not in the eRAM gold standard
Differential diagnosis candidates generated by each graph	True Positive (TP)	False Positive (FP)
Differential diagnosis candidates not generated by each graph	False Negative (FN)	True Negative (TN)

### Evaluation results

#### Metrics comparison and optimal threshold selection

**Fig. 2:**
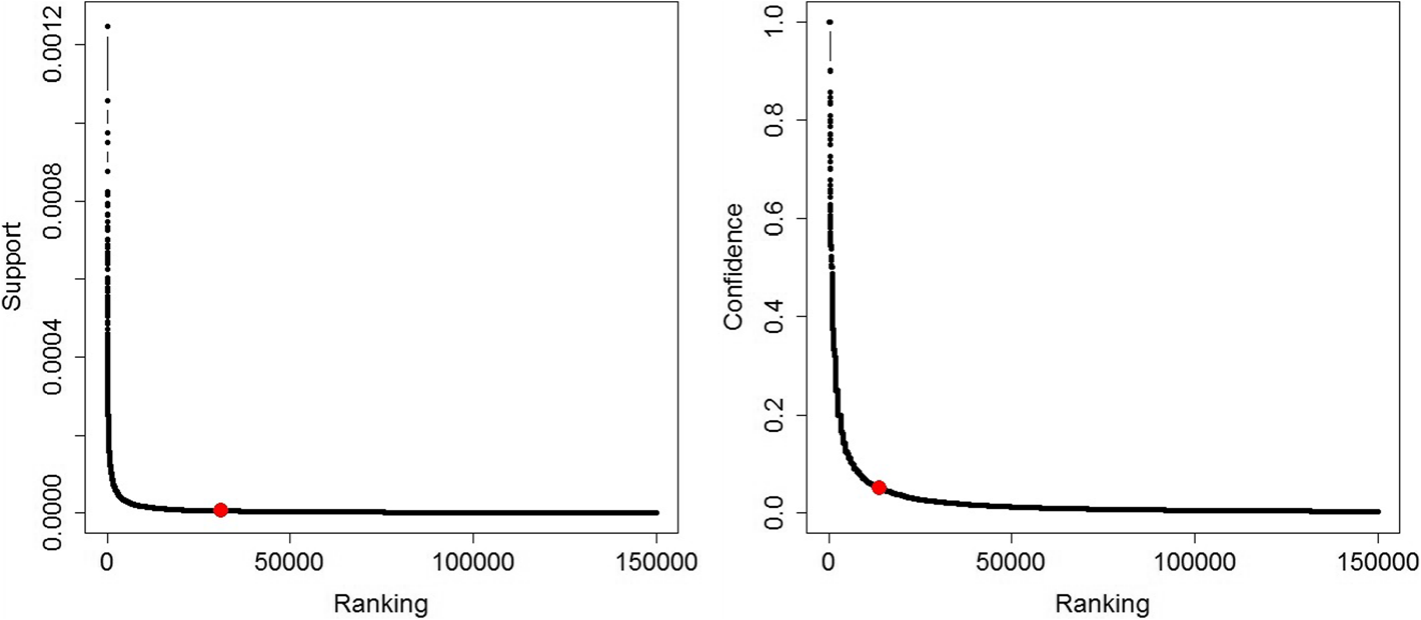
Plotted curve between association ranking and two metrics

Since HPO-Orphanet is a rare disease dominant knowledge resource, we focused on the enrichment of HPO-Orphanet with associations between phenotypes and rare diseases mined from EMR. To select the optimal associations, we set average support score 5E-06 as the threshold to first select 31,211 frequent itemsets and we then set average confidence score 0.05 as the minimum confidence to finalize 13,742 associations (see Additional file 1). To further validate the selection of thresholds, as shown in Fig. 2, we found that both support and confidence value didn’t have much fluctuation after dropping below their average values (the threshold point is marked on the curve).

**Fig. 3:**
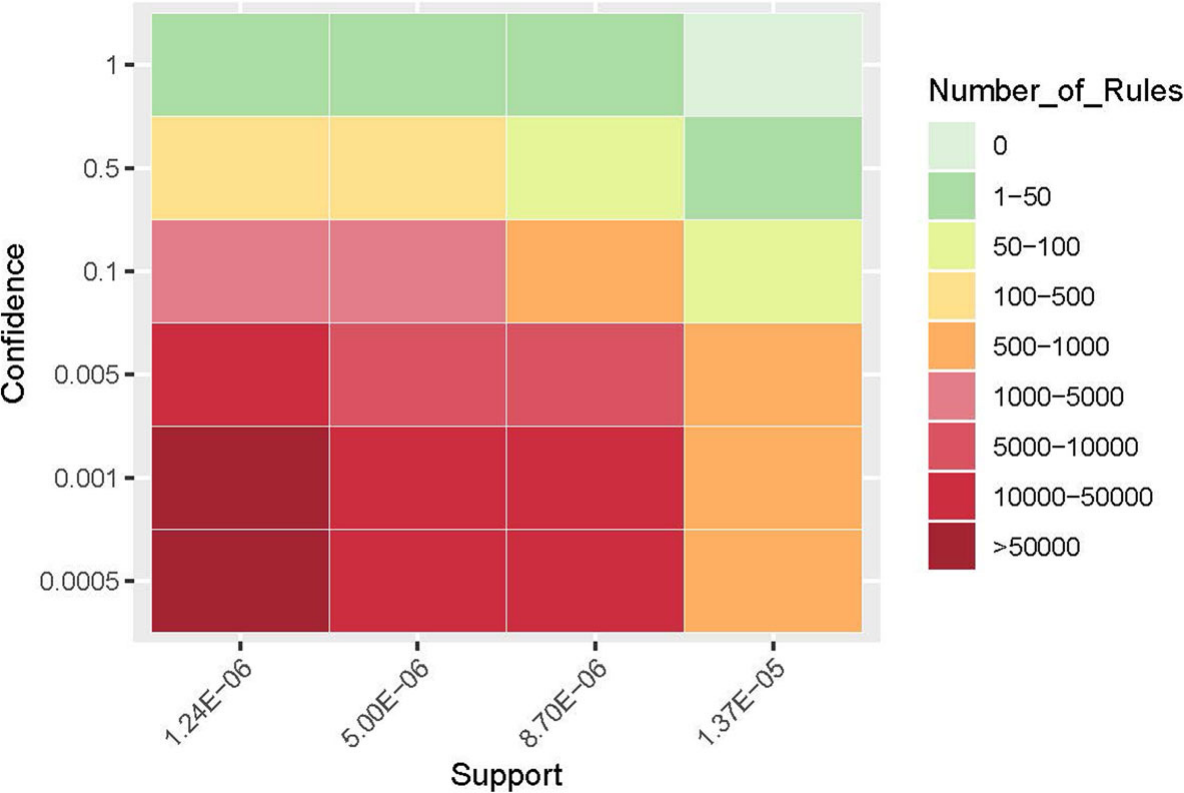
Characterization of associations

We also characterized the associations using a heatmap as shown in Fig. 3. Specifically, x-axis indicates support value ranges from 1.24E-06 to 1.37E-05 and y-axis indicates confidence value ranges from 0.0005 to 1. From Fig. 3, we observed that the number of rules get decreased with the increment of both support and confidence.

#### Enrichment of phenotype-disease associations

As shown in Table 2, 379 rare diseases, 324 phenotypes, and 1775 associations were found from EMR but not appeared in HPO-Orphanet.

**Table 2:** Statistics between EMR and HPO-Orphanet on the number of rare diseases, phenotypes, and phenotype-disease associations

Number of Unique Rare Diseases	Count
HPO-Orphanet	2664
EMR	476
HPO-Orphanet and EMR	97
In EMR but not in HPO-Orphanet	379
Number of Unique Phenotypes	Count
HPO-Orphanet	4577
EMR	1337
HPO-Orphanet and EMR	1013
In EMR but not in HPO-Orphanet	324
Number of Unique Associations	Count
HPO-Orphanet	7529
EMR	1973
HPO-Orphanet and EMR	198
In EMR but not in HPO-Orphanet	1775

10,132 and 4742 pairs of associations can be found from 124,989 associations in literature for EMR and HPO-Orphanet respectively. According to Eq. 6, IEP for enrichment of phenotype-disease associations is 4.48%, quantifying the enrichment after EMR mining.

$$ IEP=\frac{U{K}_i-U{K}_n}{U{K}_i}\ast 100=\frac{\left(124,989-4,742\right)-\left(124,989-10,132\right)}{\left(124,989-4,742\right)}\ast 100=4.48\% $$

#### Enrichment of disease differential diagnosis

##### Bipartite graph analysis

We constructed three bipartite graphs for the HPO-Orphanet, EMR, and HPO-Orphanet+ based on 97 shared diseases. As shown in Table 3, although EMR provided fewer phenotypes than HPO-Orphanet, associations between disease and phenotypes were richer, resulting in an enriched bipartite graph after combination. We also observed that EMR graph has a higher density than HPO-Orphanet graph, indicating that some phenotype-disease pairs held too many associations and imbalanced the entire graph density. The density for the HPO-Orphanet+ graph was the lowest among all graphs. The increment of average degree for combined graph indicated that novel phenotype-disease associations were mined from EMR to enrich HPO-Orphanet. According to Eqs. 7 and 8, for a given graph G, Density(G) is calculated by $\overline{\Delta }\left(\mathrm{G}\right)$/(|v|-1). Although the HPO-Orphanet+ held the highest average degree, since vertices got enriched, resulting a relative lower density for the HPO-Orphanet+.

**Table 3:** Graph characterization for bipartite graphs generated from the HPO-Orphanet, EMR, and HPO-Orphanet+ (based on 97 shared diseases)

	HPO-Orphanet Graph	EMR Graph	HPO-Orphanet+ Graph
# of Disease Nodes	97	97	97
# of Phenotype Nodes	722	670	1194
# of Edges	1973	2071	3914
Density	0.006	0.007	0.005
Average Degree	4.818	5.4	6.064

We also listed top 15 diseases with highest degrees for each bipartite graph as shown in Table 4. After combining the two datasets, we found that some diseases with highest degree mined from EMR graph were still dominant in the HPO-Orphanet+ graph, such as *multiple myeloma*, *hodgkin lymphoma*, *giant cell arteritis*, and *follicular lymphoma*. But some dominant diseases in HPO-Orphanet graph were not ranked high in the HPO-Orphanet+ graph, such as *22q11.2 deletion syndrome*, *granulomatosis with polyangiitis*, and *marfan syndrome*. In addition, we observed that *neurofibromatosis type1* is the one that didn’t appear in the top list for either EMR or HPO-Orphanet, denoting that the combination of EMR and HPO-Orphanet enriched the phenotypic sets for *neurofibromatosis type1* and thus increased its connectivity.

**Table 4:** Top 15 diseases with the highest degree in bipartite graphs generated from the HPO-Orphanet, EMR, and HPO-Orphanet+

HPO-Orphanet Graph	EMR Graph	HPO-Orphanet+ Graph
22q11.2 deletion syndrome	multiple myeloma	multiple myeloma
melas	hodgkin lymphoma	hodgkin lymphoma
granulomatosis with polyangiitis	follicular lymphoma	giant cell arteritis
marfan syndrome	giant cell arteritis	follicular lymphoma
neurofibromatosis type 1	primary sclerosing cholangitis	primary sclerosing cholangitis
trisomy 18	myasthenia gravis	22q11.2 deletion syndrome
eosinophilic granulomatosis with polyangiitis	granulomatosis with polyangiitis	granulomatosis with polyangiitis
giant cell arteritis	pulmonary arterial hypertension	melas
acromegaly	liposarcoma	myasthenia gravis
primary sclerosing cholangitis	eosinophilic esophagitis	rheumatic fever
systemic sclerosis	rheumatic fever	marfan syndrome
dermatomyositis	klatskin tumor	dermatomyositis
osteogenesis imperfecta	tetralogy of fallot	pulmonary arterial hypertension
addison disease	cystic fibrosis	craniopharyngioma
cushing syndrome	craniopharyngioma	neurofibromatosis type1

#### Rare disease differential diagnostic suggestions – Use case study

We carried a use case study on *Hodgkin lymphoma* to compare the performance for three different bipartite graphs. The number of unique differential diagnostic suggestions for *Hodgkin lymphoma* generated by HPO-Orphanet, EMR, and HPO-Orphanet+ is 2663, 10,064, and 11,439 respectively.

**Fig. 4:**
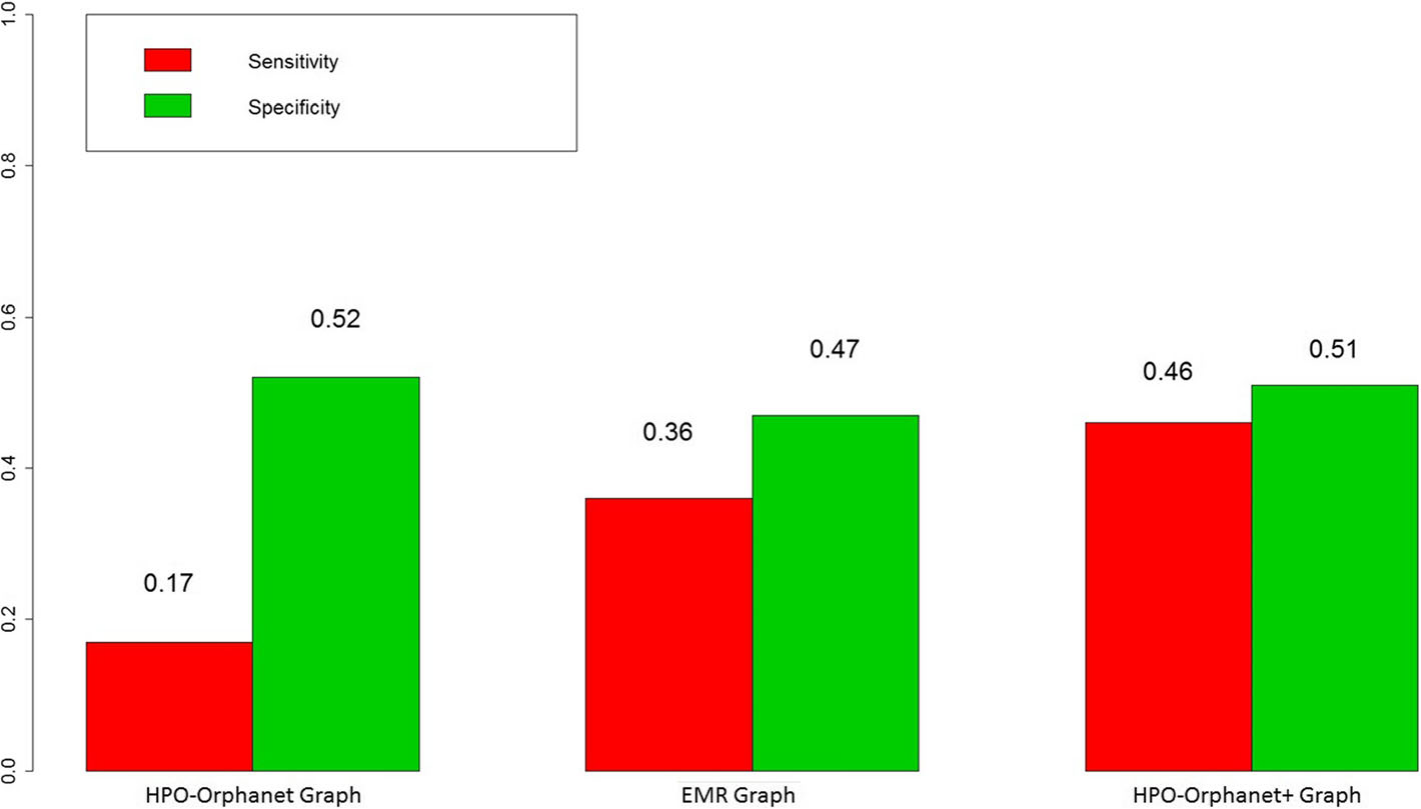
Comparison on differential diagnostic suggestion performance for Hodgkin Lymphoma

**Fig. 5:**
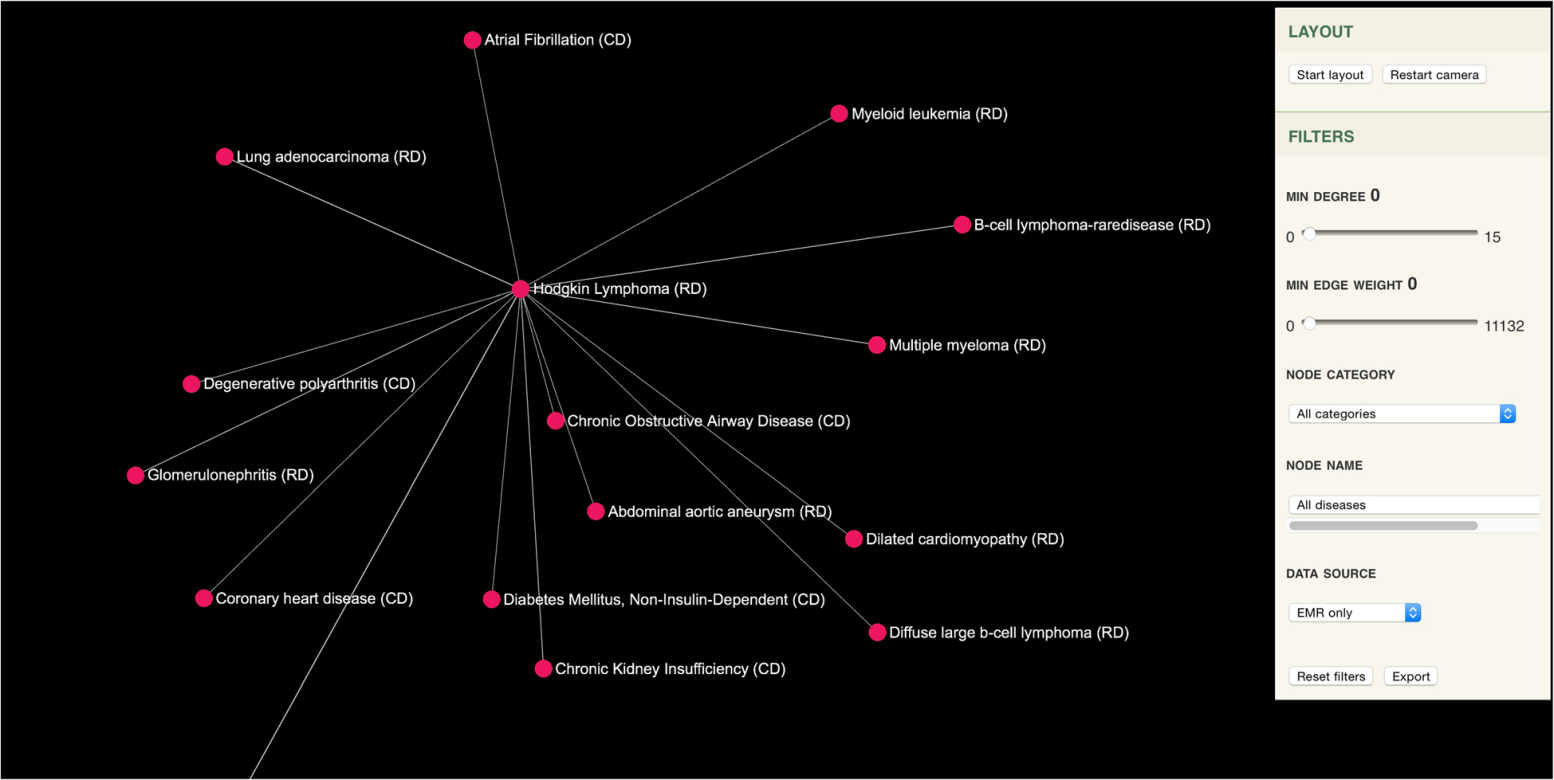
Interactive web-based tool for differential diagnostic suggestion (CD stands for Common Disease, and RD stands for Rare Disease)

*Hodgkin lymphoma* is a type of *lymphoma* that results from white blood cells called lymphocytes. Common symptoms related to *Hodgkin lymphoma* are *painless swelling of lymph nodes in neck*, *armpits or groin*, *persistent fatigue*, *fever and chills*, *night sweats*, *rapid weight loss*, *itching*, *increased sensitivity to the effects of alcohol* [39]. Sensitivity and specificity for generating differential diagnostic suggestions for *Hodgkin lymphoma* with different graphs is shown in Fig. 4. The HPO-Orphanet+ graph shows the highest sensitivity for detecting the right similar diseases according to the eRAM gold standard, while using the HPO-Orphanet graph yields the lowest sensitivity. In addition, specificity does not show significant differences among three graphs, indicating that all of them have similar performance on rejecting non-relevant diseases for *Hodgkin lymphoma*. In general, we observed that the HPO-Orphanet+ graph enriched the existing rare disease knowledge resources and thus be able to provide better diagnostic suggestions. A web-based tool was implemented to visualize diagnostic suggestions and Fig. 5 shows an example of this differential diagnostic decision aid interface by considering *Hodgkin lymphoma* as a center node.

Table 5 shows top 15 differential diagnostic candidates for *Hodgkin lymphoma* between the HPO-Orphanet+ graph and two existing diagnostic tools (Phenomizer and eRAM). The HPO-Orphanet+ graph identified 46.7% (7 out of 15) common diseases and 53.3% (8 out of 15) rare diseases. Specifically, *chronic obstructive airway disease*, *diabetes mellitus*, *atrial fibrillation*, *glaucoma, coronary heart disease*, *degenerative polyarthritis*, and *chronic kidney insufficiency* are common diseases that share the most similar phenotypes with *Hodgkin lymphoma*, which are considered to be potential candidates for misdiagnosis of *Hodgkin lymphoma*. In addition, based on literature and online material review, we found that 10 out of 15 diagnostic candidates were proved to be strongly associated with *Hodgkin lymphoma* based on similar comorbidities or complications [5, 40–48]. For example, *lung adenocarcinoma* has similar characterizations with *Hodgkin lymphoma* [46]*,* and *glomerulonephritis* is a well-recognized complication of Hodgkin disease [49]. Few evidences were detected for *dilated cardiomyopathy*, *abdominal aortic aneurysm*, *degenerative polyarthritis*, *atrial fibrillation*, and *glaucoma* from online materials and scientific literature, indicating that the associations mined from patients’ data provided new evidences for differential diagnosis of Hodgkin lymphoma.

**Table 5:** Top 15 differential diagnostic candidates for the HPO-Orphanet+ graph, Phenomizer, and eRAM on Hodgkin lymphoma. Scores in column 1 and 3 indicate Jaccard similarity and scores in column 2 indicate the IC-based score calculated by the Phenomizer (CD stands for common disease, and RD stands for rare disease)

HPO-Orphanet+ Graph	Phenomizer	eRAM
B-cell lymphoma (RD): 0.626	Classic hodgkin lymphoma (RD): 3.986	Nodular lymphocyte predominant hodgkin lymphoma (RD): 0.458
Diffuse large b-cell lymphoma (RD): 0.62	Behcet syndrome (RD): 3.189	Schnitzler syndrome (RD): 0.273
Chronic Obstructive Airway Disease (CD): 0.595	Aggressive systemic mastocytosis (RD): 3.176	Mantle cell lymphoma (RD): 0.25
Dilated cardiomyopathy (RD): 0.594	Alveolar echinococcosis (RD): 3.085	Pulmonary blastoma (RD): 0.25
Abdominal aortic aneurysm (RD): 0.592	Systemic lupus erythematosus (RD): 2.997	Aggressive systemic mastocytosis (RD): 0.22
Glomerulonephritis (RD): 0.591	Legionellosis (RD): 2.878	Anemia, autoimmune hemolytic (RD): 0.219
Diabetes Mellitus, Non-Insulin-Dependent (CD): 0.588	Takayasu arteritis (RD): 2.731	Hughes syndrome (RD): 0.219
Multiple myeloma (RD): 0.588	Cystic echinococcosis (RD): 2.648	Follicular lymphoma (RD): 0.214
Atrial Fibrillation (CD): 0.585	Eosinophilic granuloma (RD): 2.647	Thymic carcinoma (RD): 0.214
Glaucoma (CD): 0.58	Whipple disease (RD): 2.638	Mast cell sarcoma (RD): 0.2
Myeloid leukemia (RD): 0.58	Familial thrombocytosis (RD): 2.632	American trypanosomiasis (CD): 0.2
Coronary heart disease (CD): 0.58	Systemic mastocytosis (RD): 2.622	Alpha-heavy chain disease (RD): 0.194
Degenerative polyarthritis (CD): 0.573	Emberger syndrome (RD): 2.549	Klatskin tumor (RD): 0.192
Lung adenocarcinoma (RD): 0.572	Hypocomplementemic urticarial vasculitis (RD): 2.548	Legionellosis (RD): 0.189
Chronic Kidney Insufficiency (CD): 0.571	Babesiosis (RD): 2.499	Babesiasis (RD): 0.182

While differential diagnostic candidates provided by the Phenomizer are all rare disease. Similarly, the eRAM generates 93.3% (14 out of 15) rare diseases but only 6.7% (1 out of 15) common diseases.

Since many rare diseases are commonly misdiagnosed as common diseases, it is essential to link common and rare diseases at the early time of diagnosis to assist in diagnostic decision support. Compared to the Phenomizer and eRAM, the HPO-Orphanet+ graph is more capable of detecting such associations.

## Discussion

Our system can benefit the clinical practice by continuously mining knowledge from EMR to make a enriched rare disease knowledge resource incorporating information from both knowledge and data-driven insights, which is currently lacking in other systems [50–52]. However, false positive phenotype-disease relationships contributed by comorbidities are hard to detect. The odds ratio can address this issue to some extent. We will extend our current singleton frequent item set association rule mining to include two items and three items (e.g., bigram and trigram) to better support elimination of false positives. In addition, according to some existing and our previous studies [53–56], we also set thresholds as the average of metrics to select optimal associations. In the future, we will make an optimal threshold selection scheme combining both average value and elbow criterion [57] in association rule mining. Moreover, for those novel disease-phenotype associations mined from data and cannot be validated by biomedical literature, online database or knowledge base, we will recruit domain experts to provide a manual evaluation and curate the enriched knowledge base in the future work. More evaluation metrics (e.g., precision, recall, and F-measure) will be applied based on experts’ judgements.

In this study, we extracted the co-occurrence information between a phenotype and a disease from diagnosis section contained in clinical notes. Specifically, we first split the entire notes into sentences and then applied the aforementioned annotation pipeline on each sentence. In addition, problems in those documents are generally itemized entries as either phrases (e.g., *Allergic rhinitis/vasomotor rhinitis*) or short sentences (e.g, *Her asthma appeared to be very mild*), therefore, we didn’t use window size to limit the distance between phenotype and disease. In the future, to generalize the association mining on larger size of documents, we will seek to investigate the selection of appropriate window size for a better performance [58]. Moreover, some network analysis approaches [59] with knowledge network discovery algorithms [60, 61] will be incorporated with association rule mining to reveal hidden relations among diseases.

We used the SemMedDB to measure the IEP of knowledge enrichment. However, some evidences indicated that the SemMedDB is not so accurate due to the limitation of the extraction algorithms used. For example, the SemRep (the generator for SemMedDB) yielded about 75% precision on information extraction [62]. In the future, we will incorporate more disease and phenotype knowledge bases with human annotated associations to measure the knowledge enrichment.

We compared the HPO-Orphanet+ with both the Phenomizer and eRAM in this study on differential diagnostic suggestions. Results showed that the HPO-Orphanet+ is capable of providing a diagnostic graph mixed with both rare and common diseases, which has potential usage in rare disease differential diagnosis, especially for those rare diseases sharing similar symptoms with common diseases. In the future, we will upgrade the HPO-Orphanet+ by mining disease-gene information from literature [11, 63]. In addition, one recent research proposed a novel idea by introducing the concept of “property” as a third layer in addition to traditional two-layer disease-phenotype relationship [64]. This study was able to calculate the probability of getting specific diseases from a multisymptom Naïve Bayes algorithm. The third layer of “property” or multisymptoms is an interesting concept that may be involved in our future work.

## Conclusions

In this study, we proposed a data-driven approach to mine phenotype-disease associations buried in EMR so as to enrich current rare disease knowledge with newly extracted associations as well as differential diagnostic suggestions.

## Additional file

Additional file 1: Top Associations. This file includes 13,742 top phenotype-disease associations selected by support and confidence. (XLSX 218 kb)

## Abbreviations

CD: Common Disease
EMR: Electronic Medical Record
EP: Explanatory Power
FN: False Negative
FP: False Positive
GARD: Genetic And Rare Diseases
HPO: Human Phenotype Ontology
HPO-Orphanet: Original HPO-Orphanet knowledge resource
HPO-Orphanet+: Enriched HPO-Orphanet knowledge resource by EMR mining
IEP: Increment Of Explanatory Power
NLP: Natural Language Processing
OMIM: Online Mendelian Inheritance In Man
RD: Rare Disease
SemMedDB: Semantic Medline Database
TN: True Negative
TP: True Positive
UMLS: Unified Medical Language System
